# Paraneoplastic Erythema Annulare Centrifugum Eruption (PEACE) Associated With Metastatic Colorectal Adenocarcinoma

**DOI:** 10.7759/cureus.18443

**Published:** 2021-10-02

**Authors:** Chase A Pitchford, Mary E R. Shenk, Susan Zurowski, Emily H Smith

**Affiliations:** 1 Department of Dermatology, University of Missouri, Columbia, USA

**Keywords:** clinical dermatology, annular erythema, paraneoplastic syndrome, pathology, cancer and oncology, erythema and urticaria/figurate erythemas, cutaneous signs of disease

## Abstract

Erythema annulare centrifugum (EAC) is a figurate erythema presenting with annular, erythematous plaques with a trailing scale. It is considered a hypersensitivity reaction to a variety of antigens and is associated with multiple underlying causes including malignancy. Malignancy-associated EAC is termed paraneoplastic erythema annulare centrifugum eruption (PEACE). Although a specific etiology has yet to be elucidated, this form of EAC is likely to occur due to cytokine and/or antigen stimulation from an underlying malignancy. PEACE is primarily associated with lymphoproliferative disorders and rarely solid tumors. Our report discusses a case of PEACE associated with metastatic colorectal adenocarcinoma. On presentation to our clinic, the patient had developed a migratory annular eruption over a year. His review of symptoms was positive for signs of underlying malignancy, including weight loss and recent lower vertebral fracture. A biopsy of his annular lesion revealed a non-specific pityriasiform dermatitis. A vertebral biopsy uncovered a diagnosis of metastatic colorectal cancer. At that time, clinicopathologic correlation allowed us to reach the diagnosis of PEACE.

## Introduction

Erythema annulare centrifugum (EAC) is a chronic reactive form of annular erythema that appears as an urticaria-like papule and enlarges centrifugally, then clears centrally [1,2]. It is thought to be a type IV hypersensitivity reaction to various conditions, including infections, drug exposures, food exposures, autoimmune disease, and malignancy [2,3]. It can also be idiopathic. Rarely, EAC can occur in a paraneoplastic setting termed paraneoplastic erythema annulare centrifugum eruption (PEACE) [4]. Herein, we report a case of PEACE associated with metastatic colorectal adenocarcinoma.

## Case presentation

**Figure 1:**
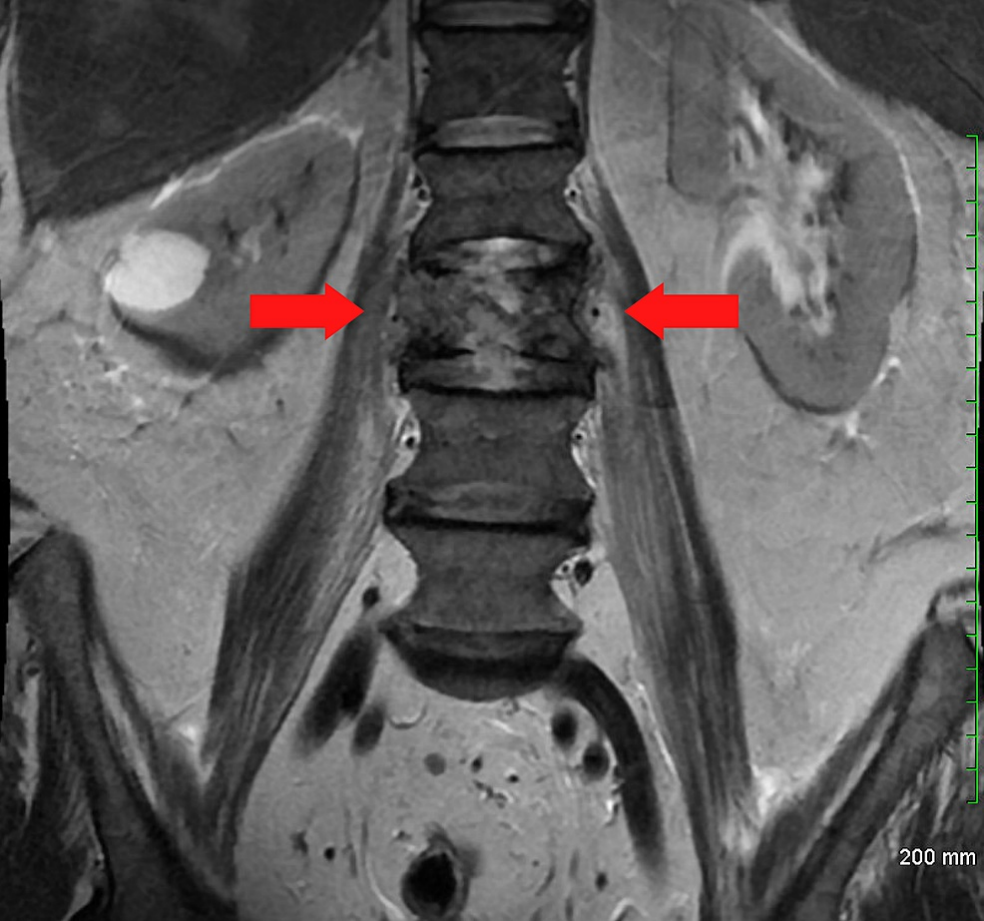
MRI of lumbar spine. Aggressive marrow replacing a mass of the L3 vertebral body causing focal severe spinal canal narrowing. Pathology showing metastatic poorly differentiated carcinoma. Red arrows indicate the mass effect in the vertebral body as a result of metastatic disease.

**Figure 2:**
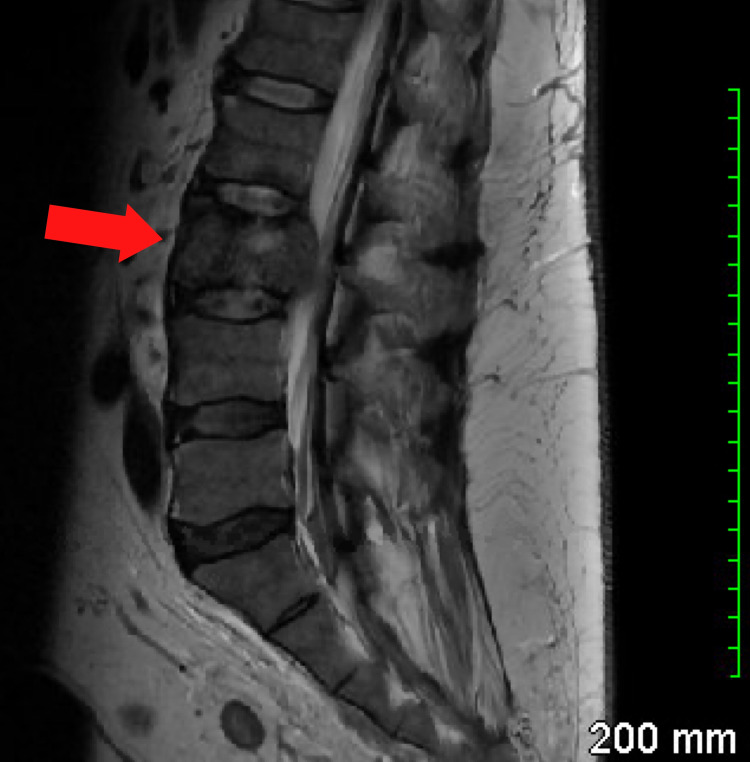
MRI lumbar spine (sagittal view). Aggressive marrow replacing a mass of the L3 vertebral body causing focal severe spinal canal narrowing. The red arrow indicates the mass effect in the vertebral body as a result of metastatic disease.

A 53-year-old man presented to our dermatology clinic for evaluation of an asymptomatic skin eruption of approximately one-year duration. The eruption started on his abdomen and then progressed to his back and bilateral extremities. The patient noted that the lesions would slowly “migrate” to new places on his body. The review of symptoms was significant for 60-70-pound weight loss over the past three months and acute onset back pain thought to be related to a work injury. He noted intermittent rectal bleeding and chart review showed no colorectal cancer screening. A review of a recent MRI revealed concern for a malignant fracture of the L3 vertebrae (Figure 1 and Figure 2).

**Figure 3:**
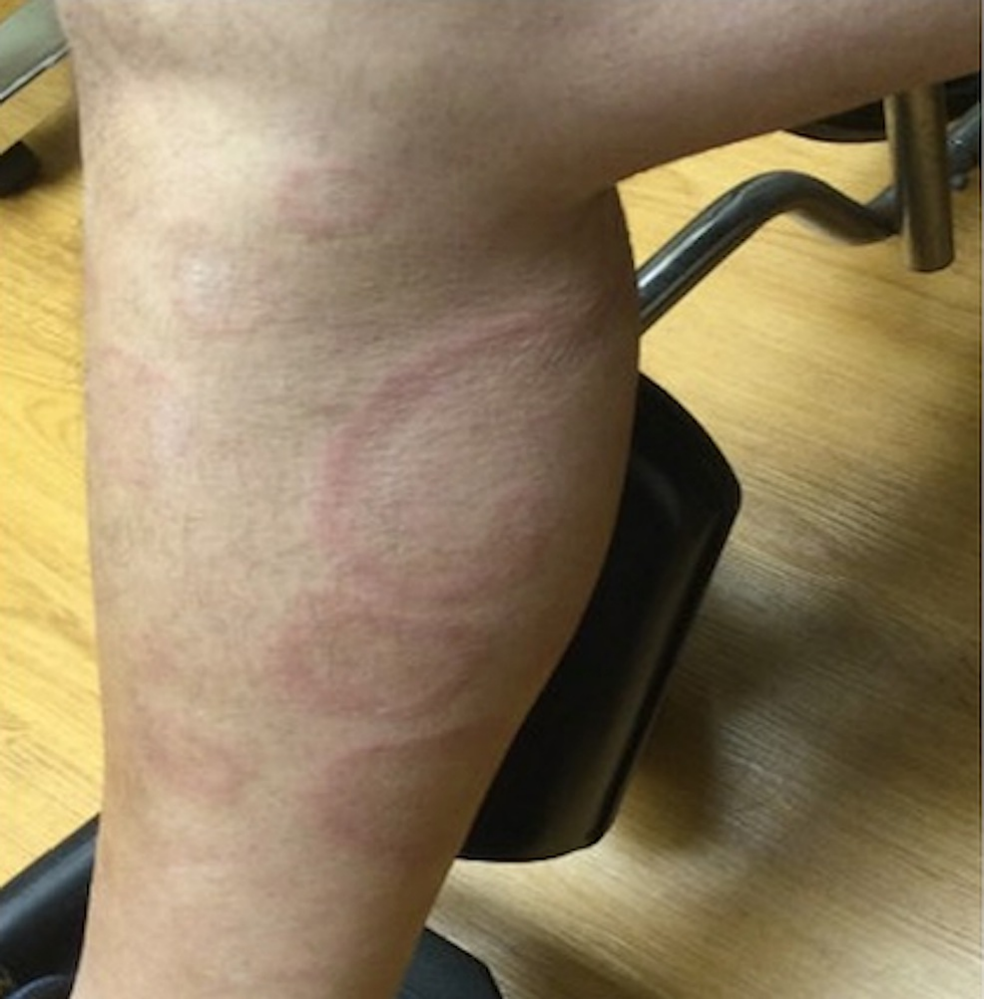
Right lower leg. Erythematous annular plaques with trailing scale on the right lower leg.

**Figure 4:**
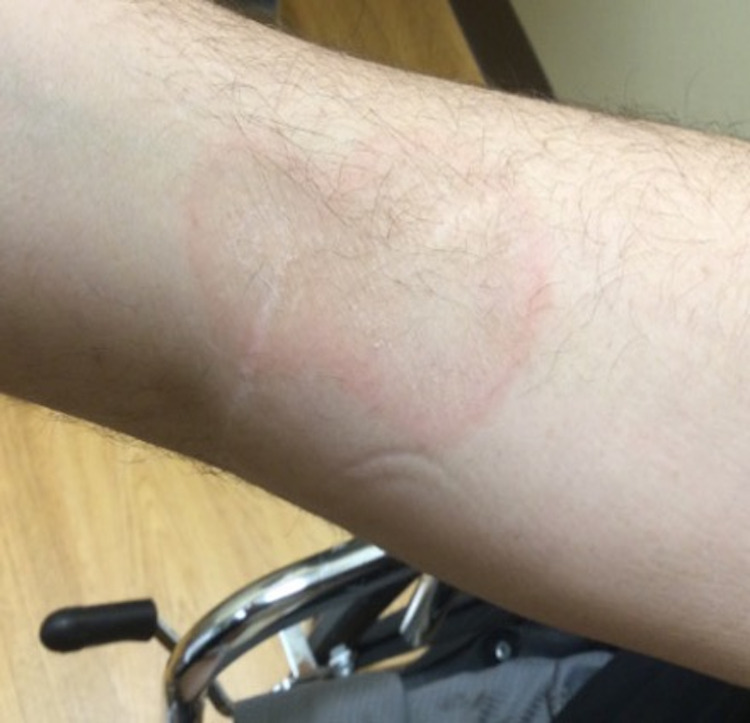
Right forearm. Erythematous annular plaques with trailing scale on the flexor surface of the right forearm.

Physical examination revealed multiple erythematous annular plaques with central clearing and trailing edge of scale on the abdomen, back, and bilateral upper and lower extremities (Figure 3 and Figure 4).

**Figure 5:**
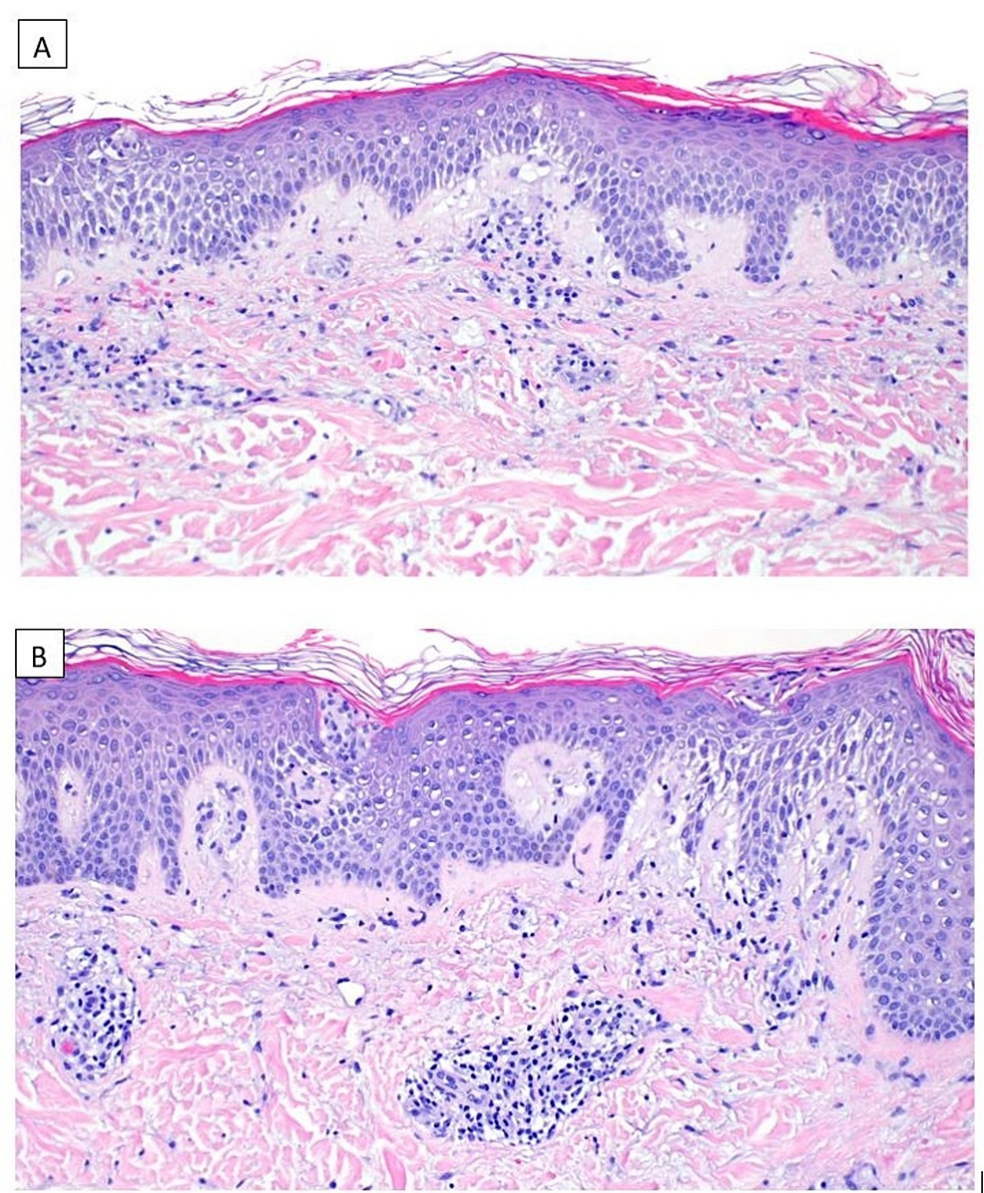
Histopathological features. Sections display mounding parakeratosis with marked spongiosis, extravasated erythrocytes, and rare Langerhans microabscesses. (A) Hematoxylin and eosin, original magnification ×10; (B) hematoxylin and eosin, original magnification ×20.

Potassium hydroxide (KOH) preparation was negative for fungal elements. A punch biopsy of one of the lesions showed pityriasiform dermatitis (Figure 5).

Clinicopathologic correlation allowed for a diagnosis of EAC. A core needle biopsy of his L3 vertebrae revealed poorly differentiated metastatic adenocarcinoma, with pathology consistent with primary colorectal disease, and thus a diagnosis of PEACE was made. The patient underwent a dedicated workup for suspected underlying malignancy. A CT-guided core needle biopsy of his L3 vertebral body demonstrated metastatic poorly differentiated adenocarcinoma. The neoplastic cells were reactive for cytokeratins AE1/AE3 and cytokeratin 20 (CK20) and non-reactive for cytokeratin 7 (CK7), compatible with a gastrointestinal primary. Next-generation sequencing detected the following genetic aberrations: adenomatous polyposis coli E (APCE 1309), Kirsten rat sarcoma viral oncogene homolog (KRAS G12C), mutY DNA glycosylase (MUTYH G382D), and phosphatidylinositol-4,5-bisphosphate 3-kinase catalytic subunit alpha (PIK3CA G1049R). A staging positron emission tomography (PET) scan identified an fluorodeoxyglucose (FDG)-avid non-uniform circumferential mural thickening of the lower rectum associated with perirectal lymphadenopathy, consistent with rectal cancer. Additional hepatic, lung, and skeletal metastasis were seen. The patient began radiation treatment with subsequent folinic acid, fluorouracil, oxaliplatin (FOLFOX) chemotherapy with Avastin.

## Discussion

EAC is a rare reactive erythema associated with annular, erythematous plaques with an associated trailing scale. EAC associated with underlying malignancy, termed PEACE, is exceedingly rare [2]. The exact pathophysiological correlation between the metastatic disease and EAC is yet to be understood, but cytokine and/or antigen stimulation in the development of the dermatoses has been proposed [5]. A case series discussed forty cases of PEACE and found 62.5% associated with lymphoproliferative disorders, most commonly leukemia and lymphoma, with 37.5% being solid tumors [5]. Of solid tumors associated with EAC, only three were associated with underlying gastrointestinal tumors [5]. Understanding the potential role of EAC in paraneoplastic processes is significant as it could indicate the presence of a previously undiagnosed malignant process. 

Clinically, there are several other annular eruptions in the differential diagnosis of EAC, making clinicopathologic correlation essential for diagnosis. EAC has been historically split into superficial and deep subtypes, with many similar but distinct histopathological characteristics. Our case is consistent with the superficial variant. Although it is commonly taught that the infiltrate of both deep and superficial EAC is “tightly cuffed” around blood vessels, this finding is more suggestive of the deep variant [6]. In fact, a case series of the histopathologic features of EAC found only one “tightly cuffed” perivascular infiltrate identified in fifty cases of superficial EAC [6]. Superficial variants are classically present with marked spongiosis, parakeratosis, Langerhan cell microabscesses, crusts, and edema of the papillary dermis, which can histologically resemble nummular dermatitis, pityriasis rosea, and allergic contact dermatitis [6]. Pityriasis rosea is particularly difficult to differentiate histologically from superficial EAC; a comparison performed between thirty-three cases of pathologically diagnosed pityriasis rosea and superficial EAC noted no significant differences between the two [6]. Despite this, clinical distinction between EAC and pityriasis rosea is easily made, as in our case.

## Conclusions

EAC is thought to arise from a type IV hypersensitivity reaction to a variety of conditions. Clinically, the lesion resembles other annular erythemas, and clinicopathological correlation is important for making a diagnosis. When a diagnosis of EAC is made, it is essential to consider the entire clinical picture as the annular eruption can arise in the setting of paraneoplastic disease. Based on histologic characteristics, there are two primary variants of EAC, superficial and deep. Moreover, the superficial variant resembles pityriasis rosea, with current literature showing no significant differences histologically. Due to their histologic resemblance, it is of utmost importance to clinically distinguish the two as we did in our case. 
